# AdmixKJump: identifying population structure in recently diverged groups

**DOI:** 10.1186/s13029-014-0031-1

**Published:** 2015-02-03

**Authors:** Timothy D O’Connor

**Affiliations:** Institute for Genome Sciences, Program in Personalized and Genomic Medicine, Department of Medicine, University of Maryland School of Medicine, 801 W Baltimore St, Baltimore, 21201 MD USA

**Keywords:** Admixture, Population genetics, 1000 Genomes project, Fine scale population structure

## Abstract

**Motivation:**

Correctly modeling population structure is important for understanding recent evolution and for association studies in humans. While pre-existing knowledge of population history can be used to specify expected levels of subdivision, objective metrics to detect population structure are important and may even be preferable for identifying groups in some situations. One such metric for genomic scale data is implemented in the cross-validation procedure of the program ADMIXTURE, but it has not been evaluated on recently diverged and potentially cryptic levels of population structure. Here, I develop a new method, AdmixKJump, and test both metrics under this scenario.

**Findings:**

I show that AdmixKJump is more sensitive to recent population divisions compared to the cross-validation metric using both realistic simulations, as well as 1000 Genomes Project European genomic data. With two populations of 50 individuals each, AdmixKJump is able to detect two populations with 100% accuracy that split at least 10KYA, whereas cross-validation obtains this 100% level at 14KYA. I also show that AdmixKJump is more accurate with fewer samples per population. Furthermore, in contrast to the cross-validation approach, AdmixKJump is able to detect the population split between the Finnish and Tuscan populations of the 1000 Genomes Project.

**Conclusion:**

AdmixKJump has more power to detect the number of populations in a cohort of samples with smaller sample sizes and shorter divergence times.

**Availability:**

A java implementation can be found at https://sites.google.com/site/igsevolgenomicslab/home/downloads

## Introduction

Correctly identifying population structure is important both to understand population history and to mitigate potential confounding signals in association analyzes in molecular epidemiology [1]. Recent population divisions can be statistically difficult to recognize as there has not been substantial time for the groups to differentiate. Objective methods to identify recent population divisions are needed. STRUCTURE [2] was the first to do this, but its Bayesian framework is not computationally efficient with whole genome sequence data. ADMIXTURE [3] has implemented a cross-validation approach to select the correct number of K (i.e. clusters or putative populations) [4], but how this statistic performs on recent population divisions with realistic simulations has yet to be evaluated. Also, alternative approaches may be better suited to recent demographic events. In this paper, I present an implementation and adaptation of the “jump method" of Sugar and James [5] for the problem of identifying populations in genomic sequence data and termed this method the AdmixKJump approach and compare its performance with the cross-validation approach.

## Method

To better facilitate ease of use and comparison with ADMIXTURE, I have implemented AdmixKJump in java with input files that can be taken directly from ADMIXTURE’s output. Parameters can be estimated from different sources (e.g. STRUCTURE or ADMIXTURE) and then used here to identify the number of clusters.

The method makes use of an information-theoretical approach where the distortions for any given K (*d*_*K*_) can be calculated using the mean squared error between the genotypes and those predicted by the admixture model:

(1) $$ \hat{d}_{Ki} = \frac{1}{M}\times \sum_{l=1}^{M}\left(\left[2\sum_{k=1}^{K} \hat{p}_{lk}\times \hat{q}_{ki}\right]-g_{il}\right)^{2}  $$

where for individual *i* we sum across all *M* markers (typically single nucleotide variants) indexed by *l*, and *K* clusters indexed by *k*. *g* represents the genotypes in the form 0, 1, or 2; $\hat {p}$ represents the estimated allele frequency for a specific cluster; and $\hat {q}$ represents the modeled proportion of each individual to each cluster and is usually interpreted as the ancestry percentage [2,3]. $\hat {d}_{K}$ is calculated as the average of $\hat {d}_{\textit {Ki}}$ for all *N* individuals. Note, Sugar and James [5] originally formulate the *d*_*K*_ value with the Mahalanobis distance, but simplified it to the mean squared error because of the complexity of calculating the covariance matrix. I have made the same simplification in this implementation.

The jump statistic (*J*_*K*_) is a measure to identify the “elbow” in the monotonically decreasing values of $\hat {d}_{K}$ as the value of *K* increases. As per Sugar and James [5], it is calculated using a transformation value *Y* as:

(2) $$ J_{K} = \hat{d}_{K}^{-Y}-\hat{d}_{K-1}^{-Y}  $$

and the estimated number of clusters (*K*^∗^) is selected by:

(3) $$ K^{*} = \arg \max_{K} J_{k}  $$

The transformation value shifts the focus to a particular part of the $\hat {d}_{K}$ distortion curve, e.g. smaller values bias towards a lower *K*^∗^. To mitigate the subjectivity of selecting *Y*, I modified the selection of *K*^∗^ by estimating the lowest possible value of *Y* that would select for a given K (*Y*^∗^[*k*]). *K*^∗^ is then selected by taking the largest value of *K* where *Y*^∗^[*K*+1]−*Y*^∗^[*K*]>0, or in other words the largest *K* where some value of *Y* supports its selection. This is the metric I evaluate in simulation and with real data.

## Testing

An important parameter space for these methods is recent population splits such as within continent population divisions. Accordingly, I test how each method fares in identifying the correct number of clusters as a function of time. I use a coalescent simulation framework based on realistic parameters from the Exome Sequencing Project [6,7] to generate whole genome sequences (i.e. 3,000 megabases) with two populations. I used these parameters with the coalescent simulator MSMS [8] to generate the data. The perl script that runs these parameters and imputes them to MSMS can be found in the program distribution. One extra parameter is added which allows me to vary the split time between two populations. This value ranged from 0 to 50K with 50 replicates for every 2K years. Sample size per population was also set to 10, 30, and 50 (20 and 40 not shown, but are consistent). The generated data was subsequently filtered with linkage-disequilibrium pruning and the removal of all singletons as is typical in admixture analysis [2,3]. I evaluated the accuracy of AdmixKJump and cross-validation by the number of times it correctly identified *K*^∗^=2.

I then apply both metrics to pairwise European populations of the 1000 Genomes Project [9]. I filter individuals, sequenced using SOLiD technology which add additional structure to the data from technical artifacts (see Figure S4 of [9] for evidence of this effect). I also excluded the Spanish population, as after filtering, only six samples remained. I then LD pruned the remaining data. This resulted in over 600K single nucleotide variants for 347 samples from 4 populations.

## Results and discussion

In simulation, I find that population structure signals evaporate after the exponential expansion in human population size at about 5 thousand years ago (KYA). One potential explanation for this lack of signal is the reduced effect of genetic drift due to increased population size. With a sample size (*N*) of 50 for each of two populations, AdmixKJump reaches 100% accuracy at 10KYA, whereas the cross-validation metric obtains 100% power at about 14KYA. The data generated with the test demographic model produce data with an average F _*ST*_ of 0.009 for 10KYA and 0.015 for 14KYA.

**Figure 1:**
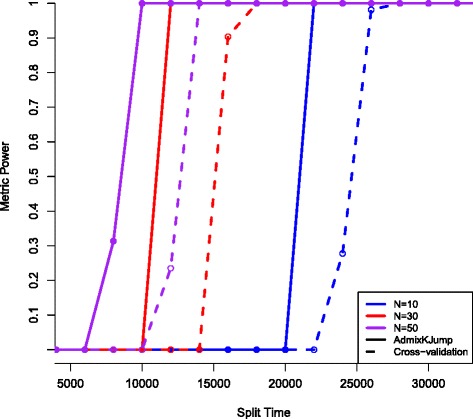
**Split time vs metric accuracy.** The x-axis is a split time parameter added to the Human demographic model indicating the point when two populations start diverging. The y-axis has two labels, the first, Ancestry Accuracy, indicates how accurate the model parameters correctly cluster the two populations, where 50% accuracy is a random assignment. The second y-axis label indicates the % accuracy of AdmixKJump or cross-validation to correctly identify *K* ^∗^=2 or two clusters. I am reporting population sample sizes of 10 (blue), 30 (red), and 50 (purple).

I also find that the new measure has more power with smaller sample sizes, for instance *N*=30 is 100% at 12KYA for AdmixKJump (see Figure 1).

With the 1000 Genomes populations, the cross-validation approach identifies *K*^∗^=1 for all pairs. The AdmixKJump algorithm finds similar results for all comparisons except the Finnish/Tuscan pair, where it finds *K*^∗^=2, consistent with the known population bottleneck of the Finnish population [10], the greatest geographic separation within these populations, and larger F _*ST*_ values (see Table 1). Thus, for closely related populations AdmixKJump is more sensitive.

**Table 1:** **European 1000 genomes project pairwise comparison for F **_***ST***_ **and** ***K*** ^**∗**^

**Pair**	**F**_***ST***_	***K*** ^**∗**^
CEU-FIN	0.006	1
CEU-GBR	0.002	1
CEU-TSI	0.003	1
FIN-GBR	0.005	1
FIN-TSI	0.009	2
GBR-TSI	0.004	1

AdmixKJump shows two clusters for one of the pairwise comparisons (FIN-TSI) whereas cross-validation does not. This is consistent with the increased divergence of this pair compared to the others, which here is measured by F_*ST*_.

## Conclusions

I have developed a new and powerful approach to classify population structure. I evaluated the current standard metric for large scale data sets, cross-validation, and found both in simulations and 1000 Genomes data that AdmixKJump is more powerful in recently diverged populations and with smaller sample sizes.

## Abbreviations

KYA: Thousand years ago
